# Leaders of ASCO, ASH, ASTRO, and NCCN Embrace Collaboration With Advanced Practitioners

**DOI:** 10.6004/jadpro.2014.5.2.8

**Published:** 2014-03-01

**Authors:** Susan Reckling

**Affiliations:** Susan Reckling has been a freelance medical writer and editor for more than 20 years, most of them specializing in oncology. Prior to that, she served as executive editor for two monthly medical journals

## Report from JADPRO Live

This past January, nearly 250 advanced practitioners assembled at the first annual JADPRO Live educational symposium in St. Petersburg, Florida, hosted by the Journal of the Advanced Practitioner in Oncology (JADPRO). Leaders from four prominent oncology organizations championed collaborative practice as not only good for patients but for practitioners as well. Representatives of the American Society of Clinical Oncology (ASCO), the American Society of Hematology (ASH), the American Society for Radiation Oncologists (ASTRO), and the National Comprehensive Cancer Network (NCCN) offered their perspectives on the role of advanced practitioners (APs) in oncology in the interdisciplinary care of patients with cancer during a roundtable panel discussion moderated by JADPRO Editor in-Chief Pamela Hallquist Viale, RN, MS, CNS, ANP.

"To continue the approach of the past will not work in the future," predicted Robert W. Carlson, MD, who is Chief Executive Officer of NCCN. The demands are changing, and the oncology treatments available are too complicated for the current work force to deliver, he added.

With the well-known ASCO Workforce Study (ASCO, 2007) projecting a significant shortfall of oncologists and medical providers in oncology by the year 2020, the panel clearly acknowledged the expanding role of the AP in filling that gap. "Training programs are not going to make up for the shortfall projected in hematology," remarked Steven L. Allen, MD, FACP, of the Monter Cancer Center, Hofstra North Shore–LIJ School of Medicine, Lake Success, New York, who is also Chair of the ASH Committee on Practice. In fact, 25% of practicing hematologists will retire in the next 5 years, based on the responses to a September 2013 survey of ASH’s practice-based members.

## Educational Initiatives

"All of us on the panel are on the same page regarding the value of collaborative practice, but our professional organizations may be at different levels of maturation in this area," admitted Louis B. Harrison, MD, FASTRO, of Mount Sinai–Beth Israel, Mount Sinai–Roosevelt, and Mount Sinai–St. Luke’s Hospitals, New York, who is Past President and Past Chairman of ASTRO.

As cancer has evolved into a chronic disease model, the interdisciplinary medical team approach—involving oncologists, advanced practitioners pharmacists, physician assistants, physical therapists, social workers, and nutritionists (among others)—has been supported by ASCO, remarked Peter P. Yu, MD, of Palo Alto Medical Foundation, who is President-Elect of ASCO. "As a society, there is no question in our minds that to deliver high-quality care for patients with cancer, we need to embrace the view of collaborative practice," he added.

With an emphasis on education, ASCO has collaborated with many organizations, including the European Society for Medical Oncology and the National Board of Medical Examiners, to implement educational programs for advanced practitioners in oncology. For instance, mirroring their tools for fellows, ASCO launched a digital comprehensive program for newly graduated APs that focuses on team care for oncology patients, symptom management, genetics, and skills for communicating with patients. "Many advanced practitioners do not have an oncology background, so this is a very useful tool," added Ms. Viale. In addition, ASCO offers tumor-specific educational modules.

To foster awareness and interest among APs in hematology, ASH provides hematology-specific educational resources for advanced practitioners. ASH offers joint educational tools for APs, works with oncology nursing societies, and adapts courses for both advanced nurse practitioners and physician assistants. Furthermore, APs are encouraged to join ASH’s grassroots network to become involved in online advocacy campaigns. For the upcoming 2014 ASH Annual Meeting, a lunch program concerning how to incorporate advanced nurse practitioners and physician assistants into a hematology/oncology practice is being considered, added Dr. Allen.

As for NCCN, its best practices committee has conducted surveys focusing on APs among its 23 member institutions, and there is no question that advanced practitioners are of value, reported Dr. Carlson. The traditional roles of care are changing, he added, and we need to be the messengers of change rather than resist it.

At the heart of a shared team approach and agreement of standards are the NCCN guidelines, according to Dr. Carlson. "They are the generally accepted best clinical practice guidelines across the continuum of care in the world of medicine—period," he pronounced. Not only do these guidelines define an evidence-based accepted system of delivery of care, they can facilitate the expansion of care to new professional groups.

**Table 1:**
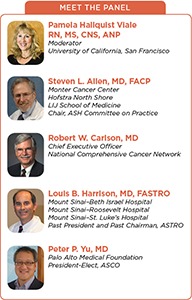


Dr. Carlson noted that many challenges facing the oncology care team are more systems-oriented than educative in nature. "We need to reconfigure how we care for patients and make that care more efficient, so the workforce available will be adequate to service their needs," he noted.

As for ASTRO, Dr. Harrison admitted that his society has been a little "slower to the punch" with respect to navigating a course with APs. He acknowledged a disconnect in their perception of the workforce among radiation oncologists, practice managers of radiation oncology, and APs. However, there is a growing awareness at ASTRO that the only way to embrace the future is through collaborative practice, and ASTRO and ASCO are working together on the workforce issue.

"Cancer patients identify with advanced practitioners more than anyone," noted Dr. Harrison, "and we need to use that unique relationship to build patient satisfaction."

## Collaborative Practice in Action

Some of the oncologists on the panel shared their own positive professional experiences working with APs in oncology. First, Dr. Allen noted that members of ASH are collaborating with APs both in daily practice and in clinical research. In fact, in his own practice (which includes 32 physicians and 16 nurse practitioners/physician assistants), the responsibilities of APs center on direct patient care under a collaborative agreement with a physician, supervision, and clinical management of treatment infusion suites, and assistance in managing data for clinical trial programs.

"Our program could not function and maintain its high standards without the assistance of our advanced practice colleagues," he stated.

As for Dr. Harrison, he has worked collaboratively with an advanced practitioner for his entire career. "I have not spent a day in my life as a radiation oncologist without an advanced practitioner partnering with me," he revealed. In fact, in Dr. Harrison’s program, the advanced practitioners are known as "the gurus" of toxicity management. "We turn to them for help as the protectors of patient safety."

Finally, Dr. Carlson revealed that he worked closely with one nurse practitioner for 18 years, another nurse practitioner for 17 years, and a physician assistant for 7 years in his outpatient clinic. "You need to set the standards incredibly high and insist on excellence," he explained. "Every one of my advanced practitioners is empowered to question anything I do at any time, and they have on many occasions." Being able to trust an AP to triage patients quickly and appropriately is essential, added Dr. Carlson.

## Research on the Value of APs

Research is clearly needed to assess the expanding role of APs on the oncology care team. Dr. Carlson mentioned the necessity of benchmarks for assessing their productivity and how many patients they should be seeing. Thus, health-care delivery research should be considered legitimate oncologic research, added Dr. Harrison.

Both Drs. Harrison and Carlson noted that the team role may not be the same for every institution. "Guidelines may be similar among institutions, but the pathways of delivering that care may be different among institutions," explained Dr. Harrison. It may be a matter of finding "the sweet spot" for how best to use the skill set of an AP within the team model, concluded Dr. Carlson.
